# Accurate pancreas segmentation using multi-level pyramidal pooling residual U-Net with adversarial mechanism

**DOI:** 10.1186/s12880-021-00694-1

**Published:** 2021-11-12

**Authors:** Meiyu Li, Fenghui Lian, Chunyu Wang, Shuxu Guo

**Affiliations:** 1 College of Electronic Science and Engineering, Jilin University, Changchun, 130012 China; 2 School of Aviation Operations and Services, Air Force Aviation University, Changchun, 130000 China

**Keywords:** Residual learning, Multi-level pyramidal pooling module, Adversarial mechanism, Pancreas segmentation

## Abstract

**Background:**

A novel multi-level pyramidal pooling residual U-Net with adversarial mechanism was proposed for organ segmentation from medical imaging, and was conducted on the challenging NIH Pancreas-CT dataset.

**Methods:**

The 82 pancreatic contrast-enhanced abdominal CT volumes were split via four-fold cross validation to test the model performance. In order to achieve accurate segmentation, we firstly involved residual learning into an adversarial U-Net to achieve a better gradient information flow for improving segmentation performance. Then, we introduced a multi-level pyramidal pooling module (MLPP), where a novel pyramidal pooling was involved to gather contextual information for segmentation, then four groups of structures consisted of a different number of pyramidal pooling blocks were proposed to search for the structure with the optimal performance, and two types of pooling blocks were applied in the experimental section to further assess the robustness of MLPP for pancreas segmentation. For evaluation, Dice similarity coefficient (DSC) and recall were used as the metrics in this work.

**Results:**

The proposed method preceded the baseline network 5.30% and 6.16% on metrics DSC and recall, and achieved competitive results compared with the-state-of-art methods.

**Conclusions:**

Our algorithm showed great segmentation performance even on the particularly challenging pancreas dataset, this indicates that the proposed model is a satisfactory and promising segmentor.

## Background

Organ segmentation from medical imaging is recognized as a difficult job, since the contours of organs tend to be indistinguishable from the background gray low-resolution images. Especially for organs with small volume and varied morphology, such as pancreas [1]. As deep learning thrives, convolutional neural networks (CNNs) show great potential on organ segmentation tasks and various methods based on CNNs have been raised for pancreas segmentation [2–14]. Zhang et al. incorporated multi-atlas registration and level-set for a pancreas segmentation framework, which mainly contained coarse, fine, and refine stages and utilized both the 3D and 2D information for segmentation [2]. Mo et al. proposed an iterative 3D feature enhancement network, which suppressed the non- target areas and increased the fine target details by leveraging the information in different layers, to segment pancreas from CT images [3]. Liu et al. raised an ensemble fully convolutional neural network for pancreas segmentation, which firstly generated candidate region and then combined output probability maps from five segmentation networks with different energy functions [4]. Roth et al. presented an automated holistically-nested convolutional network for pancreas localization and segmentation from 3D CT scans [5]. Zhou et al. put forward a fixed-point model for pancreas segmentation in abdominal CT scans through shrinking the input region by a predicted segmentation mask [6]. Cai et al. introduced a recurrent neural network to address the contextual learning and segmentation consistency problem and applied a novel Jaccard Loss to optimize training for improving pancreas segmentation performance [7]. Oktay et al. showed an attention gate model which is capable of automatically focusing on targets in medical imaging. The involvement of this attention gate into models conduces to suppressing irrelevant regions while highlighting useful features for a specific task. The U-Net model trained with the attention gate performs highly beneficial performance on 3D CT scans pancreas dataset [8]. These schemes indicate that the involvement of CNNs variants is effective for pancreas segmentation task.

Segmentation methods based on 3D networks are usually time-consuming and require more advanced server configuration. However, 2D segmentation models are not adept at capturing spatial information, thus tend to be limited in final segmentation performance. To solve this issue, we proposed a multi-level pyramidal pooling residual U-Net with adversarial mechanism for accurate pancreas segmentation in this paper. For this objective, we incorporated residual learning [15] into an adversarial U-Net to optimize the gradient information flow, thus improved its segmentation performance. Then, we put forward a multi-level pyramidal pooling module (MLPP) to collect more contextual information in search of a structure with better performance. Specifically, we introduced a novel pyramidal pooling into the adversarial U-Net with residual blocks as a substitute for the conventional pooling layer. And in order to obtain finer clues, we further introduced four groups of network structures with different numbers of pooling blocks for fusing features from different scales. To verify the effectiveness and stableness of MLPP in pancreas segmentation task, we applied pooling layers with different sizes to constitute two sets of MLPP and successively tested their segmentation performance. Overall, our proposed 2D segmentation model addresses the time-consuming and high configuration requirements of 3D networks. Then, the involvement of adversarial learning offsets the spatial information loss in simple 2D segmentation networks, depending on the ability of generative adversarial network to capture high-dimensional data distributions. And the introduction of multi-level pyramidal pooling module (MLPP) helps to collect more contextual information for segmentation. These steps contribute to compensate simple 2D segmentation networks for their spatial information loss.

The structure of the remaining paper was organized as follows. Firstly, we introduced the materials and methods used in this paper. Then, we set several groups of experiments and showed the specific results. Finally, we made a discussion and a conclusion for this work.

## Methods

### Data

We evaluated our proposed model on a public Pancreas-CT dataset [16, 17], which is collected by the National Institutes of Health Clinical Center (NIH). It contains 82 contrast-enhanced abdominal CT volumes, where each scan is 512 × 512 × L and L represents slices number along the long axis. We resized the scans to [208, 224] referring to the general scopes of label. Following the training protocol in [6, 7, 18], our experiments were carried on four-fold cross validation to randomly split these 82 patients. That is, three folds of patients were used as the training dataset and the rest samples were used for testing.

### Residual U-Net with adversarial mechanism

A typical generative adversarial network [19] is consisted of a discriminator and a generator. These two networks compete with each other in a minmax two-player game, as defined in Eq. (1). And in this formula, D and G respectively represent the discriminator and the generator. D(x) refers to the probability that the input x comes from the original dataset rather than the synthetic samples.

1 $$\mathop {{\text{min}}}\limits_{G} \mathop {{\text{max}}}\limits_{D} V\left( {D,G} \right) = E_{{x\sim p_{data} (x)}} \left[ {\log D(x)} \right] + E_{{z\sim p_{z} (z)}} \left[ {\log \left( {1 - D(G(z)} \right)} \right]$$

In the training process, the objective of the discriminator is to distinguish the real images from the synthetic samples produced by the generator, no matter how semblable they are. The generator aims to produce samples as realistic as possible to cheat the discriminator. During this process, the discriminator and the generator optimize their own network simultaneously until achieving Nash equilibrium [20]. A generative adversarial network is capable of capturing high-dimensional spatial information distributions through its specific competitive mechanism. Therefore, we introduced adversarial learning into conventional U-Net to obtain an adversarial U-Net to offset the spatial information loss in simple segmentation network, thus collecting much more useful information for pancreas segmentation.

Residual network proposed by He et al. [15] is to solve the problems of the increasing training error and degrading network performance with the network deepness added. In order to optimize the gradient information flow of our adversarial U-Net to further improve its segmentation performance, we incorporated residual learning into our proposed model.

**Fig. 1:**
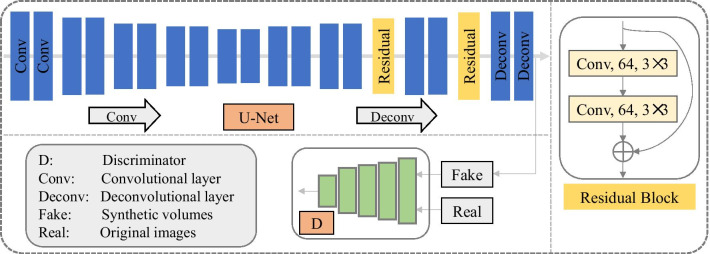
Overview of the proposed pancreas segmentation model

Specifically, the U-Net proposed by Ronneberger et al. [21] was used as a basic framework in this work. Based on this segmentation network, we involved adversarial mechanism [19] to further ensure the distributions of produced volumes resemble that of the ground truth images for a better segmentor, namely adversarial U-Net. In adversarial U-Net, a segmentation network (i.e., U-Net) is used as the generator in a generative adversarial network. Thus, the synthetic volumes produced by the generator refer to the obtained probability maps from the segmentation network. The adversarial network used here is consisted of five convolutional layers, and the kernel sizes are 7 × 7, 5 × 5, 4 × 4, 4 × 4, and 4 × 4 from the first to the fifth layer. The energy functions of the generator and discriminator in adversarial U-Net were respectively defined in Eqs. (2) and (3). *N* is the total amount of images. *F*_*n*_ and *R*_*n*_ respectively represent the fake samples from the segmentation network and the original images from the NIH Pancreas-CT dataset. *D* refers to the discriminator while *G* represents the generator (i.e., segmentor). Then, in order to further improve segmentation performance, we introduced residual learning into the adversarial U-Net, which contributes to a better gradient information flow within network. The specific structure of the involved residual block was shown in Fig. 1.

2 $$\frac{1}{N}\sum\limits_{{{\text{n}} = 1}}^{N} {\left| {F_{n} - R_{n} } \right|} + E_{{z\sim p_{\left( z \right)} }} \left[ {\log \left( {1 - D(G(z))} \right)} \right]$$

3 $$- E_{{{\text{x}}\sim p_{(data)} }} \left[ {\log D\left( x \right)} \right] - E_{{z\sim p_{(z)} }} \left[ {\log \left( {1 - D(G(z))} \right)} \right]$$

### Multi-level pyramidal pooling module

**Fig. 2:**
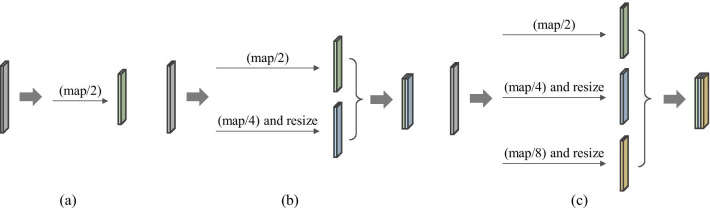
Diagram of different pooling layers. **a** Conventional pooling structure, **b** a pooling mode consisted of two scales of pooling layers, i.e., B1, **c** an escalation pooling mode consisted of three scales of pooling layers, i.e., B2

Furthermore, we introduced a multi-level pyramidal pooling module (MLPP) to collect more useful details for segmentation. Specifically, inspired by [22], we involved a novel pyramidal pooling into aforementioned segmentor (i.e., adversarial U-Net with residual blocks) for capturing contextual information in the model training process. Then, we set four groups of network structures with different numbers of pyramidal pooling blocks for fusing features from different scales, and selected the one with the optimal performance acted as the final version of our proposed algorithm. That is, in the first network, namely (i.e., B_P1), we placed one pooling block after the fourth convolutional layer. In the second network (i.e., B_P2), we placed one block after the third and the fourth convolutional layer severally. In the third network (i.e., B_P3), we placed one block after the second, the third and the fourth convolutional layer severally. In the fourth network (i.e., B_P4), we placed one block after the first, the second, the third and the fourth convolutional layer severally. To assess the robustness of MLPP for pancreas segmentation task, we severally applied two kinds of updated pooling blocks based on the conventional pooling block displayed in Fig. 2a, to constitute MLPP and successively verified their segmentation performance. The structures of these two pyramidal pooling blocks (B1 and B2) were respectively shown in Fig. 2b, c.

### Experimental environment

All experiments in this work were built on PyTorch [23] and implemented on an NVIDIA GeForce GTX 1080Ti graphics card with 11 GB memory. In the training phase, Adam was used as optimizer with initial learning rate 0.0001 and batch size was set to 1.

### Evaluation metrics

To evaluate the segmentation performance of our proposed model, we used DSC and recall as evaluation metrics in this paper. DSC reflects the overlap between the output probability maps obtained from segmentation networks and their corresponding ground truths, as defined in Eq. (4). Let X represents the voxels in obtained probability maps while Y denotes the voxels in ground truths.

4 $$DSC = \frac{{2\left\| {X \cap Y} \right\|}}{{\left\| {X + Y} \right\|}}$$

Recall measures the fractions of correctly predicted pixels in the total number of pancreas pixels, with its definition in Eq. (5) as below:

5 $${\text{Recall}} = \frac{{\left\| {X \cap Y} \right\|}}{\left\| Y \right\|}$$

## Results

### Residual U-Net with adversarial mechanism

**Fig. 3:**
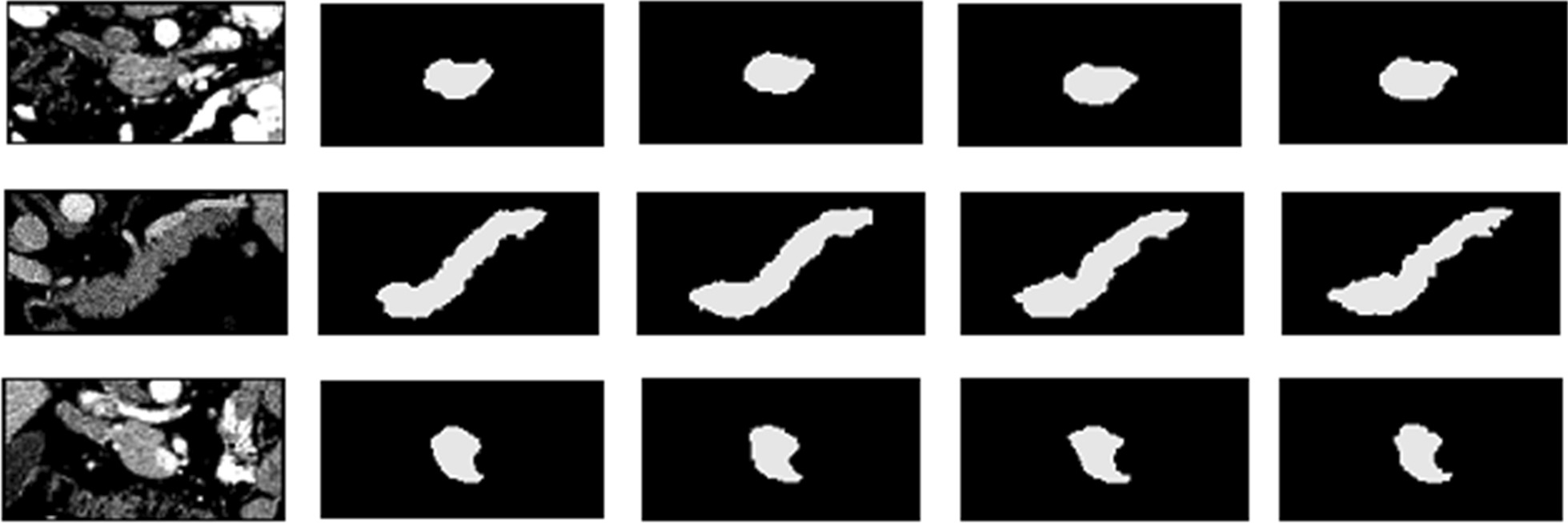
Examples of 2D visualization results from several segmentation models. From left to right, original images, U-Net results, adversarial U-Net results, results from adversarial U-Net with residual blocks, and ground truth images

In order to verify the effectiveness of residual learning, we firstly compared our proposed adversarial U-Net with residual blocks to a conventional segmentation network U-Net [21] and an adversarial U-Net. The segmentation results comparison was shown in Fig. 3. And the specific numerical values of these three models on metrics DSC and Recall were displayed in Table 1.

**Table 1:** Dice similarity coefficient (%) and recall (%) comparison of three models

	U-Net	AD-U-Net	ADR-U-Net
DSC	77.73	80.98	81.36
Recall	78.44	81.11	82.36

AD-U-Net refers to the adversarial U-Net, and ADR-U-Net represents the adversarial U-Net with residual blocks

### Multi-level pyramidal pooling module

**Fig. 4:**
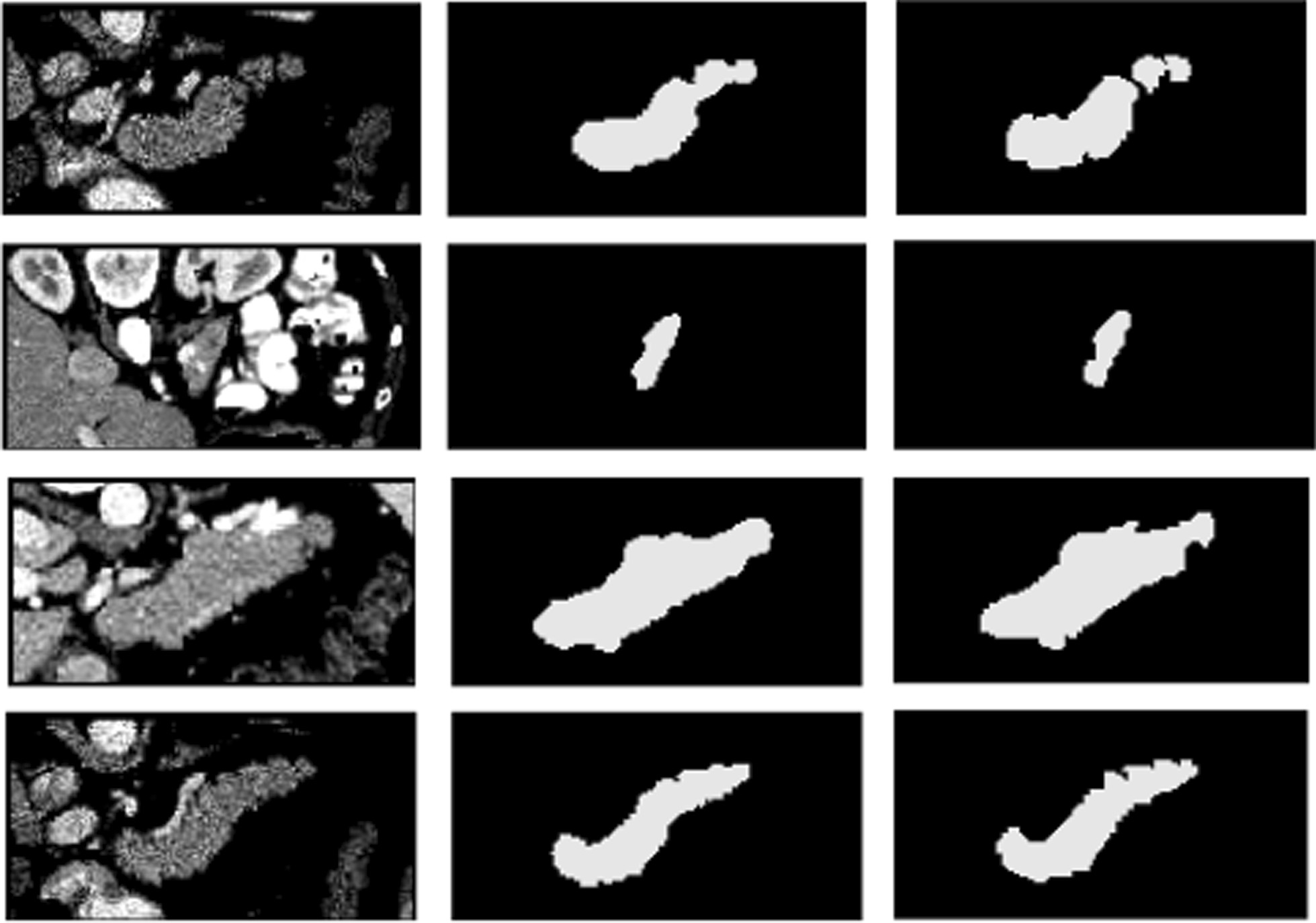
Examples of 2D visualization results from the optimal network ADR-U-Net with four pooling layers based on pooling blocks I. From left to right, original images, segmentation results, and ground truth images

**Fig. 5:**
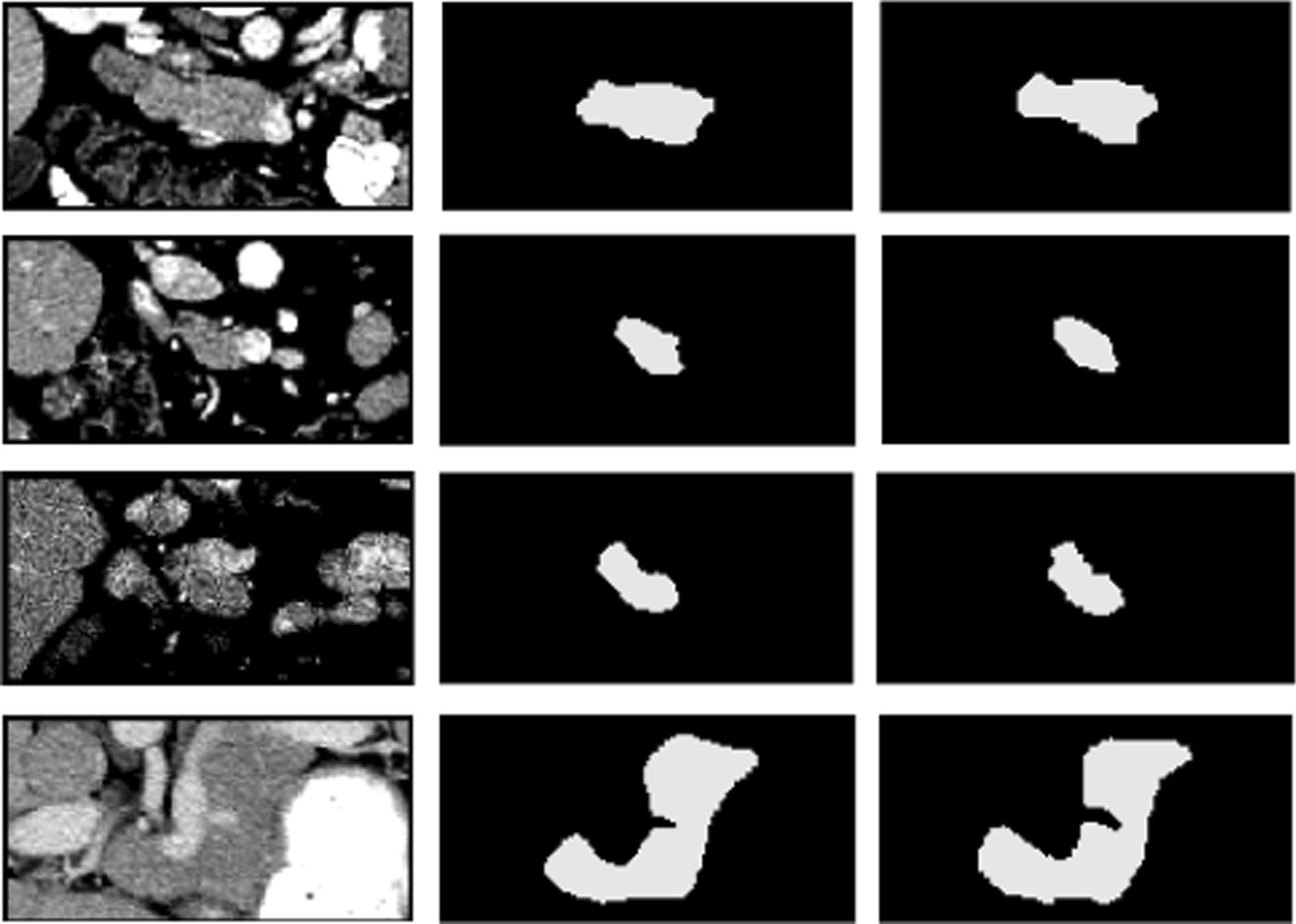
Examples of 2D visualization results from the optimal network ADR-U-Net with four pooling layers based on pooling blocks II. From left to right, original images, segmentation results, and ground truth images

In our proposed multi-level pyramidal pooling module (MLPP), we severally set one, two, three, and four pyramidal pooling blocks to obtain multi-scale feature representations, and picked out the one with optimal performance acted as the final network version. MLPP was severally consisted of two types of pooling structures: B1 and B2, as shown in Fig. 2. The specific numerical results were respectively displayed in Tables 2 and 3. Figure 4 showed the segmentation results from the optimal network ADR-U-Net with four pooling layers based on pyramidal pooling block B1 while Fig. 5 exhibited the segmentation results from the optimal ADR-U-Net with four pooling layers based on pyramidal pooling block B2.

**Table 2:** Dice similarity coefficient (%) and recall (%) comparison of ADR-U-Net models with different pooling layers based on pyramidal pooling block B1

	B1_P1	B1_P2	B1_P3	B1_P4
DSC	81.65	82.12	82.43	82.77
Recall	82.10	83.31	83.34	84.32

**Table 3:** Dice similarity coefficient (%) and recall (%) comparison of ADR-U-Net models with different pooling layers based on pyramidal pooling block B2

	B2_P1	B2_P2	B2_P3	B2_P4
DSC	82.22	82.74	82.76	83.03
Recall	83.38	83.75	84.06	84.60

### Compared with the state-of-the-art methods

To evaluate the segmentation performance of our proposed model on pancreas segmentation task, we compared it with the state-of-the-art pancreas segmentation methods. The probabilistic bottom-up approach [18] based on multi-level deep convolutional networks achieves a DSC score of 71.8%, which is 11.23% lower than our proposed method. The holistically-nested convolutional neural network proposed by Roth et al. [5] obtains a mean DSC score of 81.27% and is inferior to our method by 1.76%, while the attention gate model put forward by Oktay et al. [8] is 1.55% lower than our model. Also, our optimal DSC score in this work is 0.63% higher than the recurrent neural network architecture raised by Cai et al. [7] which addresses the contextual learning and segmentation consistency problem for pancreas segmentation, while our proposed method exceeds 0.66% on evaluation index DSC than the fixed-point model which involves a predicted segmentation mask to shrink the input region for a better performance when segmenting pancreas form abdominal CT scans in [6]. All these instances demonstrate that our proposed model is a powerful segmentor even on the tricky pancreas segmentation mission.

## Discussion

### Residual U-Net with adversarial mechanism

In this work, we proposed a multi-level pyramidal pooling residual U-Net with adversarial mechanism to achieve accurate organ segmentation, and we conducted our proposed model on a particularly challenging dataset (i.e., NIH Pancreas-CT) to assess its segmentation performance. Specifically, we firstly involved a discriminator into a conventional U-Net to obtain an adversarial U-Net, and used this structure as the baseline architecture in our proposed segmentation model. Then, we introduced residual learning into the baseline segmentor to optimize gradient information flow for improving model performance. Figure 3 shows the segmentation results from U-Net, adversarial U-Net, and adversarial U-Net with residual blocks. It is obvious that the output probability volumes from the adversarial U-Net with residual blocks preferably resemble the ground truths in a myriad of subtle parts compared with U-Net and adversarial U-Net, which illustrates that the involvement of adversarial learning and residual blocks are all advantageous to improve pancreas segmentation performance.

### Multi-level pyramidal pooling module

Furthermore, we introduced a multi-level pyramidal pooling module (MLPP) into the residual U-Net with adversarial mechanism to capture more contextual information for pancreas segmentation. Table 2 shows that B1_P4 precedes 1.12%, 0.65%, and 0.34% on DSC while 2.22%, 1.01%, and 0.98% on Recall than structures B1_P1, B1_P2, and B1_P3. This indicates that B1_P4 achieves the optimal segmentation performance compared to other three models as more contextual information are collected in B1_P4. The numerical values in Table 3 display the same trend as the Table 2, which further verify the above conclusion that B1_P4 performs the best on pancreas segmentation among these four sets of models. Thus, the structure B1_P4 is used as the final version of our segmentation model.

Compared with the state-of-the-art methods for pancreas segmentation, our proposed model shows competitive results as displayed in experimental parts. There is still room, however, for our proposed segmentation network. As our model is 1.47%, and 2.43% lower than the methods proposed in [24, 25]. Thus, future work will upgrade this existing model to quest a better segmentation performance on pancreas segmentation, and apply our model on different datasets to confront different kinds of organs segmentation tasks.

## Conclusions

In this paper, we exploited a novel multi-level pyramidal pooling residual U-Net with adversarial mechanism for organ segmentation, and operated this proposed model on a particularly challenging NIH Pancreas-CT dataset. Experimental results show that our method achieves great improvements than the basic network and obtains satisfactory performance compared with the-state-of-art segmentation models even for the particularly challenging Pancreas-CT dataset. This also indicates that our proposed segmentation model is an effective and promising network for organ segmentation.

## Data Availability

The datasets analysed during this current study are available in the public NIH Pancreas-CT dataset with a website of https://wiki.cancerimagingarchive.net/display/Public/Pancreas-CT.
